# Increased Fibroblast Growth Factor 21 (FGF21) Concentration in Early Second Trimester Amniotic Fluid and Its Association with Fetal Growth

**DOI:** 10.3390/metabo11090581

**Published:** 2021-08-28

**Authors:** Nikolaos Vrachnis, Savvas Argyridis, Dionysios Vrachnis, Nikolaos Antonakopoulos, Georgios Valsamakis, Christos Iavazzo, Dimitrios Zygouris, Nikolaos Salakos, Alexandros Rodolakis, Nikolaos Vlahos, George Mastorakos, Peter Drakakis, Zoi Iliodromiti

**Affiliations:** 1 Third Department of Obstetrics and Gynecology, Medical School, National and Kapodistrian University of Athens, Attikon Hospital, 12462 Athens, Greece; antonakopoulos2002@yahoo.gr (N.A.); zyg14@hotmail.com (D.Z.); pdrakakis@hotmail.com (P.D.); 2 Vascular Biology, Molecular and Clinical Sciences Research Institute, St George’s University of London, London SW17 0RE, UK; 3 Department of Obstetrics and Gynecology, Archbishop Makarios III Hospital, Nicosia 2029, Cyprus; argyridissavvas@hotmail.com; 4 Department of Clinical Therapeutics, National and Kapodistrian University of Athens Medical School, Alexandra Hospital, 11526 Athens, Greece; dionisisvrachnis@gmail.com; 5 Second Department of Obstetrics and Gynecology, Medical School, National and Kapodistrian University of Athens, Aretaieio Hospital, 11526 Athens, Greece; gedvalsamakis@yahoo.com (G.V.); nicolaos.salakos@gmail.com (N.S.); nikosvlahos@med.uoa.gr (N.V.); 6 Department of Gynecological Oncology, Metaxa Cancer Hospital, 18537 Piraeus, Greece; christosiavazzo@hotmail.com; 7 First Department of Obstetrics and Gynecology, National and Kapodistrian University of Athens Medical School, Alexandra Hospital, 11526 Athens, Greece; a.rodolaki@gmail.com; 8 Unit of Endocrinology, Diabetes Mellitus and Metabolism, Second Department of Obstetrics and Gynecology, National and Kapodistrian University of Athens Medical School, Aretaieio Hospital, 11526 Athens, Greece; gmastorak@med.uoa.gr; 9 Neonatal Department, National and Kapodistrian University of Athens Medical School, Aretaieio Hospital, 11526 Athens, Greece; ziliodromiti@yahoo.gr

**Keywords:** fibroblast growth factor 21 (FGF21), insulin, amniotic fluid, fetal growth, small for gestational age (SGA), fetal growth restriction

## Abstract

Altered fetal growth, either reduced or exacerbated, is associated with adverse perinatal outcomes. The underlying pathogenetic mechanisms of altered growth remain unclear. Fibroblast growth factor 21 (FGF21) and insulin are both considered to be major regulators of tissue growth and metabolism. The aim of our study was to investigate the association of second trimester amniotic fluid FGF21 and insulin concentrations with fetal growth. The amniotic fluid concentrations of FGF21 and insulin were determined in 80 cases of different fetal growth patterns (SGA—small for gestational age, LGA—large for gestational age, and AGA—appropriate for gestational age fetuses). Both peptides were found to be increased in cases of abnormal fetal growth, reduced growth velocity (SGA), or macrosomia (LGA). Specifically, FGF21 was significantly increased, as higher FGF21 levels were observed in the amniotic fluid of SGA and LGA fetuses compared with AGA fetuses (*p* < 0.05). Furthermore, the more severe the fetal smallness, the higher the FGF21 levels (*p* < 0.05). Similarly, higher insulin levels were noted in the amniotic fluid of SGA and LGA fetuses compared with those in AGA fetuses, though this was not statistically significant (*p* > 0.05). Again, the more severe the reduced fetal growth, the higher the insulin levels.

## 1. Introduction

Fetal growth velocity abnormalities (small for gestational age, SGA; large for gestational age, LGA) are associated with adverse perinatal outcomes compared with appropriate for gestational age (AGA) fetuses [1,2,3]. SGA fetuses are further classified into constitutionally small fetuses (smaller than average, but in accordance with their genetic potential) and fetal growth-restricted fetuses (FGR, failure to reach their growth potential as a consequence of extrinsic influences, due to placental insufficiency or other less common causes). The more severely affected SGA cases that are below the third centile are considered by definition as restricted and represent a significant proportion of adverse perinatal outcome cases [4,5,6]. Nevertheless, SGA fetuses are at risk for unfavorable outcomes even in the absence of abnormal Doppler studies [7,8]. The possible pathogenetic mechanisms involved remain to date largely unknown [9,10,11], although recent data strongly point to the role of defective uterine artery remodeling [12,13]. In the context of normal pregnancy, uterine spiral arteries undergo transformation from high-resistance and low-capacity vessels to low-resistance and high-capacity vessels, thereby enhancing placental perfusion and ensuring adequate blood supply to the fetus and, subsequently, normal fetal development [14]. When, however, this extravillous trophoblast remodeling process is defective, placental perfusion is limited, leading to placental ischemia and fetal growth restriction [15].

A normal concentration of growth factors and other mediators in the amniotic fluid promotes the fetal growth rate within the normal range of growth centiles for the gestational age (10th to 90th) [16,17,18,19]. Scrutiny of various molecules of the amniotic fluid can provide information on the origin of the abnormal intrauterine growth velocity and help to identify markers [20], particularly given that amniotic fluid and plasma composition are similar early in the second trimester of pregnancy [21].

Fibroblast growth factor 21 (FGF21), a member of the FGF family, plays an important regulatory role in metabolism, primarily in glucose and lipid metabolism [22]. It induces glucose uptake in adipocytes, and thereby reduces blood glucose and enhances insulin sensitivity [23]. This action, which is insulin-independent, is likely mediated through expression induction of cellular membrane glucose transporters (GLUT1) [22]. FGF21 also exerts an inhibitory action on lipolysis [24]. The liver is the primary site of FGF21 production and action. In the fasting state, hepatic expression of FGF21 provides an adaptive response through ketogenesis, fatty acid oxidation, and gluconeogenesis [25]. A very low-calorie diet has also been shown to increase FGF21 concentrations [26]. The above data point to the anabolic and protective effects of FGF21 in well-functioning cellular metabolism, a major prerequisite for normal fetal growth. Increased levels of FGF21 are found in obesity, type 2 diabetes, and impaired glucose tolerance, indicating its role in the enhancement of insulin sensitivity [27]. FGF21 tissue growth potential has also been demonstrated in a study, which attributed to this factor a role in the association between diabetes and gynecological cancer [28].

FGF21 is inversely related to growth rate in infants, and especially in preterm infants with accelerated linear growth [29]. Research data show that fetal glucose levels are significantly lower in growth-restricted fetuses in comparison with fetuses with normal growth, with a possible correlation to the degree of underlying hypoxia [30,31]. Similar findings were reported in a study that explored maternal-to-fetal blood glucose concentration gradients for the umbilical arteries and veins; these were found to correlate with the degree of hypoxia in the SGA group [31].

However, according to human fetal experimental data [32], in the early second trimester, pancreatic immaturity leading to relative unresponsiveness to glucose levels seems to be responsible for the small changes in insulin concentrations. The insulin intracellular cascade results in anabolic and mitogenic actions, such as stimulation of cellular growth, proliferation, and differentiation. It is hypothesized that insulin and insulin-related molecules constitute dominant growth-promoting factors in the rapid phase of somatic growth during late gestation, but probably not in the early second trimester [33].

The aim of this prospective observational study was to investigate the association of early second-trimester amniotic fluid FGF21 and insulin concentrations with fetal growth in the late second and third trimester. Determination of amniotic fluid concentrations of FGF21 and insulin in cases of different fetal growth patterns (SGA, LGA, and AGA) was carried out in an effort to identify a possible connection between these hormones and the opposite extremes of fetal growth, fetal growth restriction, and fetal macrosomia, and, thereby, to partially elucidate the pathophysiological mechanisms underneath fetal growth disturbances.

## 2. Results

The study sample was 31 SGA fetuses, 18 LGA fetuses, and 31 AGA fetuses (control group), all matched for gestational age, fetal sex, maternal weight, and height. SGA was defined as a birth weight less than the 10th centile for gestational age, AGA between the 10th and 90th centiles, and LGA above the 90th centile. Maternal and fetal characteristics are shown in Table 1. There was no significant difference regarding maternal weight, maternal height, maternal parity, maternal smoking, and gestational age at birth between the three groups (*p* > 0.05). There was significant difference, however, regarding maternal age (*p* = 0.01), fetal sex (*p* = 0.02), and birth weight (*p* = 0.01) between the three groups. Multivariate analysis was conducted after initial statistical analysis to assess the effect of both factors (maternal age and fetal sex).

**Figure 1:**
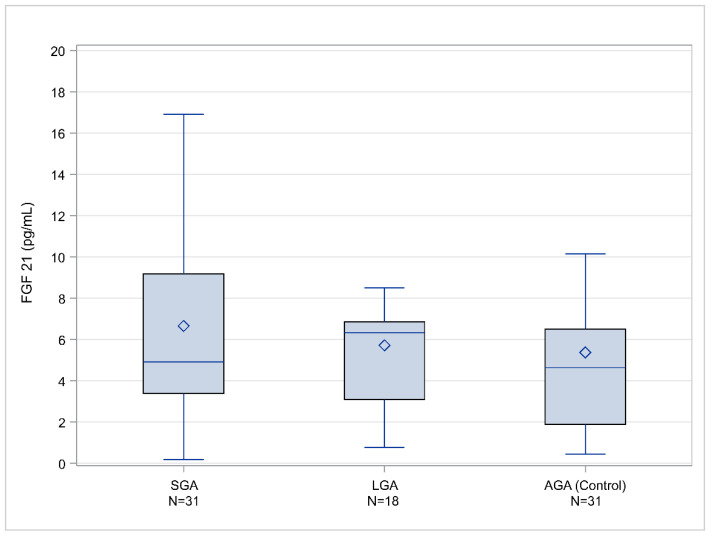
FGF21 values (pg/mL), box and whisker plots indicate box limits: Q1 and Q3, lines within the boxes: median value, diamond symbol: mean value, whisker limits: minimum and maximum observation after outlier exclusion.

Figure 1 summarizes FGF21 assay results showing statistical characteristics of FGF21 in the three studied groups (SGA, AGA, and LGA—control group). There are higher FGF21 levels in the amniotic fluid of SGA fetuses compared with those in AGA fetuses (*p* = 0.03) and LGA fetuses (*p* = 0.039), with median values of 49,060 pg/mL for SGA fetuses, 63,370 pg/mL for LGA fetuses, and 46,360 pg/mL for AGA fetuses.

To determine whether there is an association between FGF21 and the severity of fetal growth disturbances, we further analyzed FGF21 levels at the extremes of fetal weight. Table 2 presents FGF21 levels distributed by fetal size and, more specifically, the extremes of fetal size (SGA < 3rd centile, LGA > 95th centile). It was established that FGF21 levels show a progressive decrease as the SGA centiles drop. SGA fetuses below the 5th centile still display significantly higher levels of FGF21 compared with controls (though lower than those of the SGA group as a whole), while severe SGA fetuses (<3rd centile) display lower levels compared with controls (*p* < 0.01). However, it should be taken into account that the number of fetuses included in the subgroups declines correspondingly. On the other hand, severe LGA fetuses (>95th centile) manifest a further increase in FGF21 levels (*p* < 0.01), though again, the number of cases is small.

**Figure 2:**
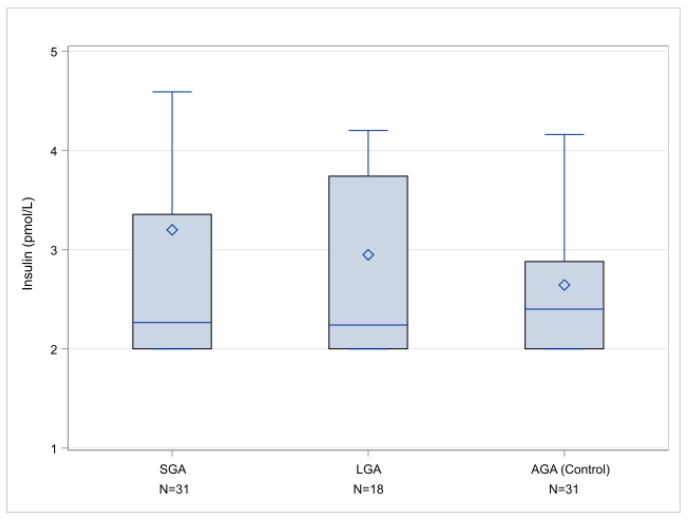
Insulin values (pmol/L), box and whisker plots indicate box limits: Q1 and Q3, lines within the boxes: median value, diamond symbol: mean value, whisker limits: minimum and maximum observation after outlier exclusion.

Figure 2 summarizes the insulin assay results, showing statistical values of insulin in the three studied groups (SGA, LGA, and AGA—control group). There are different insulin levels in the amniotic fluid of SGA and LGA fetuses compared with AGA fetuses, with median values of 2.265 pmol/L for SGA, 2.240 pmol/L for LGA, and 2.240 pmol/L for AGA fetuses. These results, however, were not statistically significant.

Table 3 presents insulin levels distributed by fetal size and, more specifically, the extremes of fetal size (SGA < 3rd centile, LGA > 95th centile).

The correlation between FGF21 and all the other arithmetic parameters (i.e., mother’s age, mother’s weight and height, parity, birth week, birth weight, birth centile, and insulin) did not show any statistical significance (Spearman correlation coefficient <0.2 in all pairs and *p* > 0.05).

Multivariate logistic regression analysis was conducted separately for groups SGA and AGA and LGA and AGA; the results are presented in Table 4. Amniotic fluid levels of FGF21 were higher in cases of pregnancies with SGA (*p* = 0.0303) and LGA (*p* = 0.0406) fetuses compared with those in controls (AGA). Additional findings showed that maternal age, female fetus, and maternal smoking were also factors contributing to SGA, albeit not to LGA. Insulin measurements yielded borderline non-statistically significant results (*p* = 0.07).

## 3. Discussion

The mechanisms underlying altered fetal growth are to date not clear, although it has been established that factors influencing fetal growth are of maternal, placental, and/or fetal origin [34,35], with maternal age in particular being well known to influence fetal growth. Other maternal factors are, inter alia, maternal size and weight, nutritional status, cigarette smoking, substance abuse, anemia, environmental exposure, and/or adequate uterine blood flow [36]. Male fetuses are heavier than females for the same day of gestation. Thus, multivariate analysis was conducted after initial statistical analysis to assess the effect of a wide range of factors, among them, notably, maternal age and fetal sex.

Among the placental factors are size, microstructure, transporters and binding proteins, nutrient utilization, and umbilical blood flow, while fetal factors include the fetus genome, hormone output, and nutrient production [37]. As the actions of the above factors and the specific contribution of each factor have not as yet been identified with clarity, several hypotheses have been proposed. Moreover, in some SGA cases, no factor can be identified, while there are LGA cases with a normal glucose tolerance test [38]. On the other hand, it is also well established that the most common cause of fetal smallness or even growth restriction is placental hypoperfusion/dysfunction, and that the more affected is the placenta, the lower centile the fetus will be and the greater the risk of an adverse outcome, especially in non-routinely monitored pregnancies [39]. It is also true that most macrosomic babies will be those of diabetic pregnancies in whom the risk of complications, such as dystocia and neonatal early metabolic dysfunction, is increased [40].

To the best of our knowledge, this is the first study in which FGF21 and insulin levels in the amniotic fluid of fetuses between 15 and 22 weeks of gestation were determined and assessed according to birth weight, in an effort to investigate their possible role in fetal growth and, thereby, to partially elucidate the pathophysiological mechanisms behind fetal growth disturbances.

It was anticipated that there would be a difference in birth weight among the studied groups given that the latter were defined based on birth weight, with SGA fetuses having, by definition, lower birth weight than average, while LGA fetuses have a higher than average birth weight. Further anticipated differences concerned maternal age, which is also, as mentioned above, a well-recognized factor influencing fetal growth and fetal sex, with male fetuses being heavier than females [34].

FGF21 was significantly increased among SGA fetuses compared with AGA and even LGA. This most interesting finding seems to point to an adaptive mechanism enhancing FGF21 availability in cases of reduced growth velocity. Similar mechanisms have been tentatively proposed regarding other growth factors in previous studies [41]. Another explanation for increased FGF21 in both growth centile extremes could be protection against oxidative stress and induction of tissue repair procedures. FGF21 is known to be an oxidative stress suppressor and a mediator of cell proliferation and tissue repair after injury [42]. In intrauterine hypoxic conditions, particularly in selected SGA cases affected by growth restriction, there is tissue dysfunction and hypoxic injury due to oxidative stress. Increased FGF21 could partially inhibit or else restore these effects. However, in very severe SGA cases, which are by definition FGR [7], this adaptation mechanism fails to be maintained and FGF21 levels fall below AGA levels. Specifically, in this severe SGA group, the lack of energy supply suppresses all anabolic mechanisms activated in cases of energy inadequacy and, as a result, anabolic regulators, like FGF21, are also down-regulated.

On the other hand, in cases of fetal macrosomia, especially when gestational diabetes is the underlying cause, FGF21 levels are expected to increase linearly as fetal growth rate is further increased, given the role of FGF21 in tissue growth and differentiation in addition to metabolism and energy availability regulation. This hypothesis is plausible and in accordance with our findings. As LGA fetuses are known to have higher FGF21 amniotic fluid levels compared with AGA and, at the extremes of distribution (>95th centile), FGF21 levels are even higher, the established data support our theory. Nevertheless, as FGF21 also exerts antioxidative actions [42], its up-regulation in cases of fetal hyperglycemia and macrosomia may offer protection against oxidative stress, which is the main pathway leading to short-term tissue dysfunction and long-term tissue damage [43,44].

Insulin was increased, but not found to be significantly altered in SGA and LGA fetuses compared with AGA. In early gestational stages, when there is low demand for nutrient uptake, insulin may stay unaltered, acting as the main factor supporting cellular metabolism. Furthermore, in the early second trimester, pancreatic immaturity and relative unresponsiveness to glucose levels seem to contribute to these mild changes in insulin concentrations, as this was observed in our study. If our amniotic fluid samples had been collected in the third trimester of pregnancy, insulin measurements would have been expected to reflect fetal growth velocity, as insulin and insulin-related molecules constitute the dominant growth-promoting factors of macrosomia during late gestation. LGA fetuses are characterized by increased insulin levels, which occurs when there is an excessive supply of nutrients, while insulin also serves as a mediator of energy deposition by controlling glucose blood levels. This mechanism is fully active in the third trimester when most cases with growth disturbances become clinically apparent. Prior to the second half of pregnancy, the latter mechanism may be only partially evident, as fetal metabolic regulation is still evolving and fetal nutritional requirements are limited. This may explain why insulin changes were not pronounced, thus showing no statistical significance in either SGA or LGA cases.

FGF21 and insulin play a crucial role in lipid and glucose metabolism and homeostasis. FGF21 induces glucose uptake in adipocytes, reduces blood glucose levels, and improves insulin sensitivity [23]. Increased FGF21 expression is found in the fasting state, gluconeogenesis, and ketogenesis [25], all related to FGR and, to an extent, to SGA, with this group also including growth-restricted fetuses; thus, a functional relationship between FGF21 activation and insulin sensitivity is hypothesized to be present.

Maternal and umbilical cord blood sampling in growth-restricted fetuses has shown that FGF21 is increased, as are VEGF (vascular endothelial growth factor) and sFlt-1 (soluble fms-like tyrosine kinase-1), the latter of which acts as a receptor to VEGF [45]. These substances are thought to be regulators of vasculogenesis (formation of new vessels) or angiogenesis (branching or widening of existing vessels) and are also increased in cytotrophoblast, syncytiotrophoblast, stromal, and endothelial cells of FGR fetuses. As FGR is mainly the consequence of placental hypoperfusion and insufficiency, FGF21 increase may be an effort to overcome the inadequate blood supply through modulation of tissue differentiation and enhanced placental angiogenesis. The existing literature confirms this hypothesis, as increased levels of VEGF and FGF are found in placental tissue of FGR fetuses compared with that of AGA fetuses [46,47]. Furthermore, the aforementioned response to abnormal placentation, when occurring in the early second trimester, reduces the risk of placental insufficiency complications, such as early onset FGR and pre-eclampsia. The above findings in a very specific group of SGA, namely FGR fetuses, have been reported by other researchers who further support our hypothesis of an adaptive, fetal-protecting, mechanism mediated through FGF21.

Lastly, concerning LGA fetuses, it can be hypothesized that, in cases of adequate nutrient availability and placental function, FGF21 levels mirror adequate fetal weight gain, whereas in cases of SGA and, likely, placental dysfunction, FGF21 reflects adaptation mechanisms mainly determined by the severity of weight reduction and the ability of the fetus to respond.

Our study has some limitations that should be taken into consideration. The number of examined cases was very small to produce statistical significance in certain SGA or LGA examined subgroups. Thus, future studies with larger SGA or LGA groups should be conducted. Another disadvantage is that our samples were only from the early second trimester amniotic fluid and not during later weeks of pregnancy, as it would be unethical to carry out amniocentesis solely for research purposes, with the procedure carrying a small risk of miscarriage or preterm birth; however, this deprived us of the opportunity to study FGF21 and insulin concentrations in later weeks of pregnancy. In particular, some factors leading to fetal growth disturbances during gestation take place during the late second and third trimester, as evidenced by abnormal Dopplers and other fetal compromise assessment tools, as in SGA cases complicated by FGR. Furthermore, other studies with additional molecules exerting similar or reverse actions to FGF21 are required so as to draw new conclusions and elucidate further the pathways of fetal growth.

To the best of our knowledge, this is the first study investigating the role of FGF21 and insulin in fetal growth with amniotic fluid measurements, which reflect fetal blood concentrations early in pregnancy. As human fetal studies are difficult to conduct, amniotic fluid samples are probably the most efficacious alternative to fetal blood and metabolic pathways of the fetal tissues, as amniotic fluid in the early second trimester has a composition similar to that of fetal serum [21]. This is also the first study providing information and hypotheses concerning the particular mechanisms behind altered fetal growth that occur during early pregnancy. The latter is of special importance given our aim to identify an early prognostic factor of these pregnancy complications.

## 4. Materials and Methods

### 4.1. Groups

We conducted a prospective observational study in accordance with the guidelines of the Declaration of Helsinki, which was approved by the Ethical Committee of Aretaieio University Hospital in Athens, Greece. Eighty amniotic fluid samples were prospectively collected and analyzed after the initiation of the study. Informed consent was obtained from all women who participated. The study sample was 31 SGA fetuses, 18 LGA fetuses, and 31 AGA fetuses (control group), all matched for gestational age, fetal sex, maternal weight, and height. SGA was defined as birth weight below the 10th centile, AGA as birth weight between the 10th and 90th centile, and LGA as birth weight above the 90th centile. Fetal birth weight and other parameters were also prospectively collected. All cases had an early second trimester (15–22 gestational weeks) amniocentesis with a normal karyotype. Indications for amniocentesis included advanced maternal age, increased nuchal translucency, first trimester screening increased risk, absent or hypoplastic nasal bone, and a history of previous fetal chromosomal defect or structural abnormality detected on ultrasound. Inclusion criteria included absence of congenital abnormalities and singleton pregnancies, while pregnancies with major congenital abnormalities or multiple pregnancies were excluded from the study. Patient details, including age, race, parity, height, weight, smoking, and prior history, were recorded via history, taken prior to the procedure. Gestational age was calculated based on the last menstruation and was confirmed by a crown-rump length measurement between 11 and 14 gestational weeks. Gestational age at birth, mode of birth, and neonatal weight and gender were also recorded. Amniocentesis was performed based on international standards and guidelines, and amniotic fluid samples were collected in pyrogen-free tubes, centrifuged, and stored frozen at −80 degrees until FGF21 and insulin determination. Based on growth centiles for each gestational age, a gestation-related optimal weight program (GROW) was used to assign each fetus measured to a centile [48].

### 4.2. Measurements

Determination of FGF21 and insulin was performed using the enzyme immunoassay (ELISA) kits quantikine human FGF21 and quantikine human insulin immunoassay, respectively, provided by R&D Systems Inc. (R&D Systems Inc., Minneapolis, MN, USA), in compliance with the manufacturer’s instructions. Sensitivity for the FGF21 assay is 8.69 pg/mL and the range is 31.3–2000 pg/mL, while sensitivity for the insulin assay is 2.15 pmol/L and the range is 15.6–500 pmol/L. Some insulin measurements failed as a result of an inadequate volume of amniotic fluid samples to measure both peptides.

### 4.3. Statistical Analysis

Given the number of samples, there was deviation from normality regarding measured variables (FGF21 and insulin), maternal characteristics (weight, height, age, and parity), and gestational characteristics (duration and growth centile). We used the Kruskal–Wallis test for comparison of concentrations of measured variables among the three groups and the Wilcoxon rank-sum when testing for differences between two groups. Moreover, for comparison of the categorical variables (such as gender and smoking) among the groups, we used the chi-square test. For the descriptive statistics, the number and percentages for qualitative variables, as well as median value, quartile 1 (Q1), and quartile 3 (Q3) values, for the quantitative variables are presented. The distribution of sample values was evaluated using the Kolmogorov–Smirnov test. Furthermore, we applied correlation analysis using the Spearman correlation coefficient between FGF21 levels and all other arithmetic parameters in order to identify relations of FGF21. Finally, in order to adjust for confounding factors such as maternal and gestational characteristics, we employed logistic regression analysis, using fetus status as the dependent variable and the studied arithmetic or categorical parameters including FGF21 as independent variables. Specifically, we applied two multivariate logistic regression analyses (either SGA vs. AGA, or LGA vs. AGA) and a forward selection approach; thus, the *p*-value to enter a variable into the model was *p* = 0.15, and to keep it in the model was *p* = 0.05. The level of statistical significance was set at a *p*-value of less than 0.05 and all tests were two-sided.

## 5. Conclusions

FGF21 is linked to abnormal fetal growth patterns from the early stage of pregnancy. Assessment of FGF21 in early second trimester amniotic fluid of SGA, AGA, and LGA fetuses clearly points to an association of its levels with altered growth velocities, SGA, and macrosomia; it could thus play an important role as a prognostic factor for associated adverse perinatal outcomes. Developments in genomics, proteomics, and metabolomics may in the future allow intervention in order to change the course of a pregnancy, preventing the evolution or reducing the severity of fetal growth disturbances.

FGF21 increase in SGA fetuses could be attributed to an adaptive mechanism to fetal smallness until severe growth restriction occurs. The latter hypothesis, if proven correct, could also explain the fact that extremely SGA fetuses demonstrate reduced rather than increased FGF21 levels. FGF21 exerts antioxidative actions and its up-regulation could be a response to oxidative stress present in both hyperglycemic and hypoxic intrauterine conditions. FGF21 increase may also be a response to abnormal placentation and hypoperfusion, as this factor is also a modulator of placental angiogenesis. The higher FGF21 levels of LGA fetuses are expected given the role of FGF21 in tissue growth and differentiation in addition to metabolism and regulation of energy availability.

Insulin levels are also higher in SGA and LGA fetuses compared with AGA; however, this difference was not statistically significant, probably owing to the early gestational phase of the investigation and fetal pancreatic immaturity and relative unresponsiveness at the stage that the samples were drawn. Thus, although the cascade of events leading to altered fetal growth may be initiated at an earlier stage, they take time to be established and reflected in amniotic fluid measurements.

This is the first study providing information on the mechanisms underlying altered fetal growth that occur during early pregnancy. These findings are of major importance given the necessity to identify any early prognostic factor for these pregnancy complications. An understanding of the pathway that leads to fetal growth disturbances would give us the opportunity to identify these fetuses early in pregnancy and, in the future, submit them to more frequent monitoring and treatment. Greater insight into this issue will allow us to time the interventions and the date of delivery more accurately, thus decreasing the perinatal morbidity and mortality associated with SGA and LGA fetuses.

## Figures and Tables

**Table 1:** Maternal and fetal characteristics of the studied groups (SGA, LGA, and AGA) (values presented as median and Q1–Q3 range and for categorical data as number of cases and percentage).

Variable	SGA (*n* = 31)	LGA (*n* = 18)	AGA (*n* = 31)	*p*-Value
Maternal age (years)	35 (32–37)	35 (32–37)	37 (36–38)	0.01
Maternal weight (kg)	61.5 (56.5–72)	60.5 (55–64)	66 (60–74)	0.15
Maternal height (cm)	167 (165–171)	166 (159–170)	168 (163.5–170)	0.60
Maternal parity	0 (0–1)	1 (0–1)	1 (0–1.5)	0.24
Maternal smoking	23 (76.7%)	17 (94.4%)	29 (93.6%)	0.13
Fetal sex female	19 (63.3%)	4 (23.5%)	11 (36.7%)	0.02
Birth week	38 (38–39)	38 (37–39)	38 (37–39)	0.11
Birth weight (gr)	3300 (3200–3510)	3870 (3690–4180)	2580 (2440–2750)	0.01

**Table 2:** FGF21 median values (pg/mL) and Q1–Q3 ranges by fetal size subgroups, including extremes of distribution.

Subgroup	*n* of Cases	Median Value (Q1–Q3)
SGA < 10th centile	31	4.906 (3.395–9.186)
SGA < 5th centile	18	4.898 (3.247–7.555)
SGA < 3rd centile	10	4.069 (2.524–6.769)
AGA	31	4.636 (1.891–6.494)
LGA > 90th centile	18	6.337 (3.088–6.852)
LGA > 95th centile	05	6.337 (3.631–8.508)

**Table 3:** Insulin median values (pmol/L) and Q1–Q3 ranges by fetal size subgroups, including extremes of distribution.

Subgroup	*n* of Cases	Median Value (Q1–Q3)
SGA < 10th centile	24	2.27 (2–3.355)
SGA < 5th centile	14	2.34 (2–4.03)
SGA < 3rd centile	08	3.46 (2.155–6.885)
AGA	27	2.40 (2–2.88)
LGA > 90th centile	17	2.24 (2–3.74)
LGA > 95th centile	04	2.44 (2.16–3.25)

**Table 4:** Results of logistic regression analysis for the prediction of fetus status from the study parameters. ref: reference.

	SGA and AGA		LGA and AGA	
Parameter	OR (95%CI)	*p*	OR (95%CI)	*p*
Maternal age	1.31 (1.05–1.65)	0.0179	0.95 (0.77–1.18)	0.64
Maternal weight	1.05 (0.99–1.1)	0.1147	0.95 (0.87–1.03)	0.2221
Maternal height	1.07 (0.93–1.21)	0.3453	0.98 (0.86–1.11)	0.7284
Fetal sex (ref: male)	4.38 (1.06–18.12)	0.0416	0.36 (0.06–2.12)	0.2568
Maternal smoking (ref: no)	8.99 (1.04–77.86)	0.0461	0.74 (0.04–12.97)	0.8335
FGF 21	1.07 (1.01–1.21)	0.0303	1.03 (0.89–1.2)	0.0406

## Data Availability

Access to the data supporting the findings of our study is by request from the corresponding author.
